# Role of hazelnut skin supplementation on plasma antioxidant status and cytokine profile in growing lambs

**DOI:** 10.3389/fvets.2024.1340141

**Published:** 2024-02-01

**Authors:** Maria Giovanna Ciliberti, Antonella Santillo, Mariangela Caroprese, Antonella della Malva, Antonio Natalello, Antonino Bertino, Marzia Albenzio, Agostino Sevi

**Affiliations:** ^1^Department of Agriculture, Food, Natural Resources, and Engineering (DAFNE), University of Foggia, Foggia, Italy; ^2^Department of Agriculture, Food and Environment (Di3A), University of Catania, Catania, Italy

**Keywords:** small ruminants, sustainability, by-products, immune responses, oxidative imbalance

## Abstract

In this study, the effect of hazelnut skin dietary supplementation on antioxidant status and cytokine profile was evaluated in growing lambs. A total of 22 male lambs at the age of 2 months, balanced for their initial live weight (15.33 ± SD 1.79 kg), were selected and allocated into two experimental groups: the control group (CON) receiving a maize-barley-based concentrated diet, and the hazelnut group (HS) receiving supplementation with hazelnut skin (150 g/kg on the dry matter) as a maize substitute for the concentrate diet. The experiment lasted for 56 days. Peripheral blood was collected at 7, 35, and 56 days of the experiment. The free radical scavenging activity using 1,1-diphenyl-2-picrylhydrazyl (DPPH) radical scavenging assay, the total antioxidant capacity assay (TAC), the reactive oxygen species (ROS), and reactive nitrogen species (RNS) were determined in plasma. The secretion of IL-1β, IL-6, and IL-10 cytokines was also determined by ELISA. The DPPH was affected by the interaction between feeding strategy and time of sampling (*p* = 0.039) with a higher level of DPPH at 7 days in the HS group than the CON group. The time of sampling affected the levels of plasma TAC (*p* = 0.016), while the ROS/RNS levels showed a higher value in the HS group (*p* < 0.001), on average. The antioxidant/oxidant index, which combines the TAC and the ROS/RNS levels, was not affected by the inclusion of hazelnut skin in the diet (*p* = 0.394). The cytokine profile showed a lower IL-6 secretion at both 35 and 56 days than at 7 days, on average. Furthermore, the feeding treatment affected the IL-1β level, showing a lower level in the HS group than in the CON group on average. Lambs from the HS group had higher IL-10 plasma levels than the CON group at 7 days of the experiment. The present data highlight an antioxidant effect and a modulatory role in the cytokine profile of HS supplementation in growing lambs.

## 1 Introduction

Nutrition is a key factor in determining livestock production quality (1), and the recent interest in the use of by-products as valuable alternative feed sources is crucial to reach more sustainable ruminant production (2). Agro-industrial by-products, especially, which are waste from agricultural crops or vegetable processing industries, represent a precious resource of bioactive compounds for feeding animals that also contribute to the reduction of the feed-food competition (3). Many studies have proven that hazelnut and its by-products are rich in phenolic compounds and exert strong antioxidant activities; consequently, their use as food, pharmaceuticals, and feed and in cosmetic industries is largely encouraged (4). The hazelnut skin (HS) by-product contains total phenolic compounds at a level ranging from 168 to 378 times higher than raw or roasted HS. Taș and Gökmen (5) found that the total phenolic content ranged from 51.9 to 203.1 mg gallic acid equivalent (GAE) per gram of hazelnut skin based on the varieties of hazelnut, and 60% of the total phenolic content is represented by flavonoids.

In animals, during normal metabolic processes, reactive oxygen species (ROS) can be generated as part of the oxidative eustress, i.e., a minimum amount of oxidant production is essential for life processes within cellular or tissue compartments, including the mitochondria, chloroplast, endoplasmic reticulum, peroxisomes, and others (6). When a condition of oxidative stress is established, an imbalance between pro-oxidants and antioxidants occurs. This causes several pathological conditions, including sepsis, mastitis, enteritis, pneumonia, respiratory diseases, and joint diseases, which are directly connected with animal production and welfare (7). Furthermore, young animals are more susceptible to diseases and oxidative stress conditions, which are mainly associated with adapting to dietary changes and reduced physiological antioxidant defense mechanisms (8). Therefore, supplementation based on nutritional components with antioxidant properties is important to sustain the metabolic and physiological processes of growing animals.

The potential value of HS as a source of polyphenols from waste, which is available for growing lambs’ feedstock, is highlighted in a number of studies (9–15). As indicated, dietary supplementation with HS improves the nutritional quality of lamb meat with a decrease in meat lipid oxidation (10) and an increase in health-promoting fatty acids, such as polyunsaturated fatty acids and vaccenic acid, without any detrimental effect on growing parameters (9). At the rumen level, the HS supplementation is capable of modifying the C18:1 t10/t11 ratio, suggesting a change in the biohydrogenation of linoleic acid (11). Moreover, after HS supplementation, the plasma proteome shows the involvement of different pathways, including that of the apolipoproteins (APOA1 and APOA4), which possess antioxidant activity and anti-inflammatory properties (12). Lambs’ meat proteome reveals that HS is able to modulate post-mortem processes linked with meat discoloration and tenderization rate (13, 14) In the study of Viola et al. (15), an improvement of the immune passive transfer to the suckling lambs is demonstrated after HS supplementation of ewes, which is capable of restoring the reduced immune competence connected to the growth. Hence, the inclusion of 15% of HS to replace an equal amount of maize in growing lambs can be an effective strategy to reduce the cost of disposal for industries (9).

Based on previous evidence, our hypothesis was that dietary supplementation with HS could be beneficial for the oxidative plasma homeostasis and immune status of growing lambs. Thus, the present study aims to understand the role of HS dietary intake on lambs’ oxidative status and cytokine profile.

## 2 Materials and methods

### 2.1 Animal and dietary treatments

The experiment was carried out at the University of Catania (37°24′35.3”N 15°03′34.9″E), as previously reported by Priolo et al. (9). The animal study protocol was approved by the Ethical and Animal Wellbeing Commission of the University of Catania (protocol code 15295). A total of 22 Valle del Belice male lambs were selected at the age of 2 months (15.33 ± SD 1.79 kg, average initial live weight) on a local dairy farm. The lambs were allocated into two experimental groups balanced on the initial live weight: the control group (CON; n = 11) was fed a maize–barley-based concentrated diet and the hazelnut group (HS; n = 11) was fed hazelnut skin (150 g/kg on dry matter) as a partial maize replacer (57.7%) of the concentrate diet (Dalma Mangimi S.p.a, Marene, Cuneo, Italy). After 5 days of adaptation to the experimental diets, lambs were individually fed using pelleted feed to avoid selection.

The quantity of hazelnut skin included was chosen according to its chemical composition for maintaining correct levels of protein and energy (9).

The dietary experiment lasted for 56 days, and animals were placed in individual pens (1.5 m^2^) with straw litter placed on the bottom and equipped with clean water and feed offered *ad libitum*. The ingredients and chemical composition of the experimental diets were reported in detail by Priolo et al. (9). Briefly, the CON diet was characterized by 25.3 g/kg DM of crude fat and 194 g/kg DM of crude protein, while the HS diet was characterized by 60.2 g/kg DM of crude fat and 222 g/kg DM of crude protein, respectively. The phenolic composition of hazelnut skin was mainly characterized by tannins, accounting for 78.3 g tannic acid equivalents/kg DM.

### 2.2 Sampling and analysis of blood

Peripheral blood samples from each lamb were collected from the jugular vein in heparinized vacuum tubes (Becton Dickinson, Plymouth, United Kingdom) at 7, 35, and 56 days of the experiment. Blood samples were centrifuged at 1500 × g for 20 min to obtain plasma and stored at −80°C until further analysis.

### 2.3 Determination of the total antioxidant capacity of plasma using 1,1-diphenyl-2-picrylhydrazyl (DPPH) radical scavenging assay

Plasma-free radical scavenging activity was tested using 1,1-diphenyl-2-picrylhydrazyl (DPPH) radical scavenging assay, as described by Janaszewska and Bartosz (16) and Giri et al. (17) with some modifications. The colorimetric reaction is based on the decolorization of DPPH methanol solution from violet/purple to yellow in the presence of antioxidants. The hydrogen atom-donating ability of the plasma was determined by the decolorization of the methanol solution of DPPH. A solution of 0.1 mM DPPH (in methanol) was prepared, and 400 μL of this solution was mixed with 400 μL of the sample solution (20 μL of plasma and 380 μL of 10 mM sodium phosphate buffer–PBS, pH 7.4). The reaction mixture was vortexed thoroughly and left for 30 min at 37°C. After incubation, the samples were centrifuged at 3000 x g for 5 min, and the supernatant was transferred to a 24-well plate for the reading at 520 nm. The absorbance of the samples at 520 nm was measured and compared with that of a reference sample containing only DPPH solution (containing methanol) and PBS as controls.

Scavenging activity was calculated with the following formula by Giri et al. (17):


$$\left[\%\mathrm{DPPH}\;\mathrm{radical}\;\mathrm{scavenging}\;\mathrm{activity}\right]=\left[\frac{A-\mathrm{Ax}}{A}\right]*100\text{,}$$

where A is the absorbance of the DPPH solution of the control (methanol or PBS) and Ax is the absorbance of the plasma mixed with the DPPH solution.

### 2.4 Determination of the total antioxidant capacity of plasma using total antioxidant capacity (TAC) assay

Plasma total antioxidant capacity (TAC) level was evaluated using the OxiSelect™ TAC Assay Kit (Cell Biolabs, Inc.), following the manufacturer’s instructions. In brief, TAC assay is based on a copper reduction reaction from copper (II) to copper (I) in the presence of an antioxidant such as uric acid. The absorbance values were read at 490 nm against the uric acid serial dilution standard curve (0–1 mM) and were directly proportional to the plasma TAC expressed as μM of uric acid equivalents.

### 2.5 Determination of plasma reactive oxygen (ROS), nitrogen (RNS) species level, and antioxidant oxidant balance (AOB) index

The levels of reactive oxygen species (ROS) and reactive nitrogen species (RNS) were determined using an OxiSelect *In Vitro* ROS/RNS Assay Kit Green Fluorescence (Cell Biolabs Inc., San Diego, CA) following the manufacturer’s instructions. The ROS and RNS species of plasma were measured using their reaction with 2′,7′-dichlorodihydrofluorescin (DCFH), which is oxidized to the highly fluorescent 2′,7′-dichlorodihydrofluorescein (DCF). Fluorescence was read with a fluorescence plate reader at 480 nm excitation/530 nm emission (CLARIOstar microplate reader, BMG Labtech, Ortenberg, Germany). Fluorescence intensity was directly proportional to the total ROS/RNS concentrations in plasma. The results were read against the DCF (1:10 scalar dilution with a concentration range of 0–10,000 nM) standard curve and expressed as 2′,7′-dichlorodihydrofluorescein (DCF) in micromoles per liter.

Redox balance parameters were assembled for the total antioxidant defenses and oxidants ratio calculation in the antioxidant oxidant balance (AOB) index, as previously applied in Ciliberti et al. (18). The AOB index represents the ratio between the plasma antioxidant capacity, calculated using the TAC method, and the level of plasma oxidation expressed by the ROS/RNS concentration was measured as follows: AOB = TAC/ROS-RNS.

In sheep, the AOB demonstrated an effective index for the evaluation of the effects of diet on oxidative stress caused by heat stress. An increase in the ratio demonstrates prompt activation of the antioxidant defense against pro-oxidant production, showing a lower risk for oxidative stress conditions. Data were normalized on day 0, and the AOB calculation was carried out individually for each lamb.

### 2.6 Determination of IL-1β, IL-6, and IL-10 cytokines by ELISA

Plasma concentrations of IL-6 and IL-1β were determined by sandwich ELISA according to Caroprese et al. (19) with some modifications, as previously reported in Ciliberti et al. (20). Briefly, an IL-6 and IL-1β sandwich was built using specific mouse monoclonal antibodies against ovine IL-6 and ovine IL-1β as capture antibodies (Clone 4B6 for IL-6 and Clone 1D4 for IL-1β, Serotec Ltd., Killington, United Kingdom) and rabbit polyclonal anti-sheep IL-6 and IL-1β antibodies as detection reagents (Serotec Ltd., Killington, United Kingdom). All the incubations were at 37°C. Optical density was measured at 450 nm wavelength. Samples were read against a standard curve obtained using a scalar dilution of recombinant ovine IL-6 (Cusabio Biotech Co., Wuhan, P.R. China) and recombinant bovine IL-1β (Kingfisher Biotech Inc., St. Paul, MN). The intra-assay coefficients of variation (CV) were 6% for IL-6 and 8.2% for IL-1β.

The ELISA for IL-10 in plasma was determined according to Kwong et al. (21) and Hope et al. (22), with modifications reported in the study of Ciliberti et al. (20). The sandwich ELISA was built using specific antibodies against bovine IL-10 as a capture antibody (anti-bovine IL-10 monoclonal antibody, Clone CC318, Serotec Ltd., Killington, UK) and biotinylated secondary anti-bovine IL-10 monoclonal antibody as a detection reagent (Clone CC320 for IL-10, Serotec Ltd., Killington, UK). The plates were read at 450 nm using a Power Wave XS microplate spectrophotometer (Biotek Instruments, United States). IL-10 data were expressed as nanograms per milliliter. The intra-assay coefficient of variation (CV) was 7% for IL-10.

### 2.7 Statistical analysis

All data variables were examined for normality distribution and then analyzed using the mixed procedure ANOVA of SAS (23). The model included the fixed effects of feeding strategy (CON and HS) and time of sampling (7, 35, and 56 days) and their interactions. Animals are included in the model as a random effect. A Tukey post-hoc test adjustment for multiple comparisons was used to compare feeding strategy, time of sampling, and their interaction. Significance was declared at a value of *p* of <0.05. A value of *p* of <0.10 was considered a tendency. All data are presented as mean ± SEM.

## 3 Results

### 3.1 Plasma antioxidant and oxidative status

**Figure 1:**
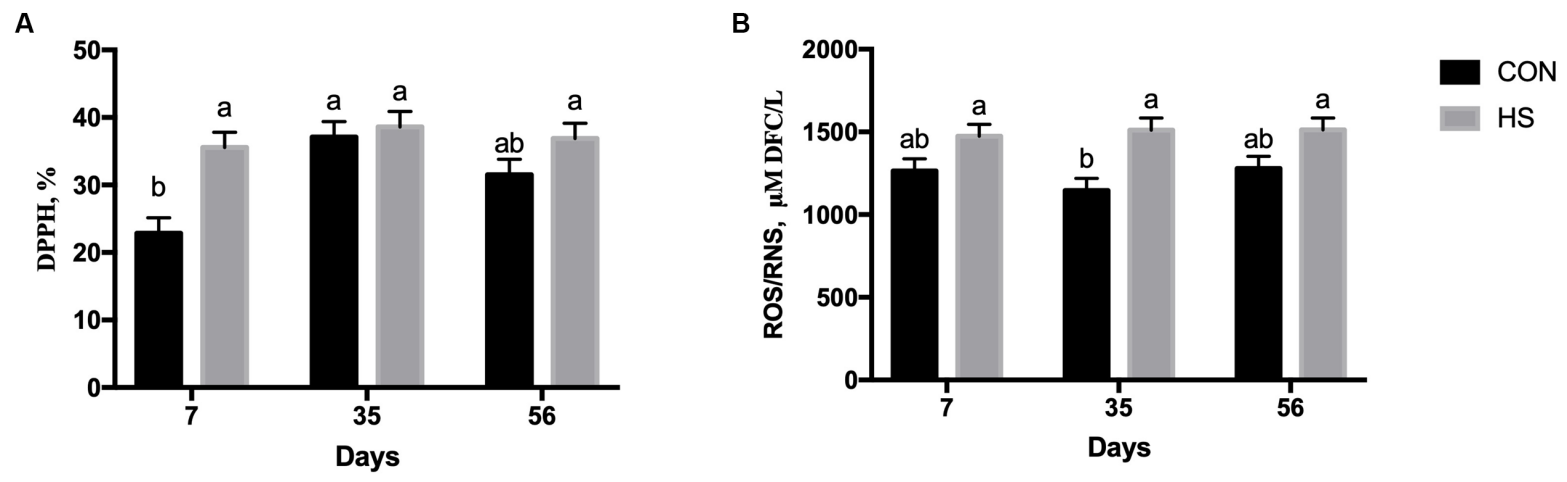
Plasma scavenging activity measured as DPPH % **(A)** and ROS/RNS production measured as micromolar of DCF/L **(B)** in lambs fed with a control maize–barley-based concentrated diet (CON) and with 15% of hazelnut skin (HS) as a maize replacer of the concentrate diet at 15, 35, and 56 days. a,b indicate significant differences among feeding treatments and time of sampling (*p* < 0.05 for DPPH, and *p* > 0.10 for ROS/RNS, respectively).

Plasma free radical scavenging activity (DPPH) was affected by feeding strategy (*p* = 0.0005), time of sampling (*p* = 0.0008), and the interaction between time of sampling and feeding strategy (*p* = 0.039). On average, the HS group showed a higher DPPH value than the CON group (p = 0.0005, Table 1), and the level of DPPH at 35 days was higher than that at 7 days of the experiment (*p* = 0.0008, Table 1). The CON group had a lower level of DPPH at 7 days than the HS group, and it was also lower than those found within the CON group at 35 days (Figure 1A).

**Table 1:** Level of the antioxidant and oxidative status biomarkers evaluated by plasma scavenging activity (DPPH %), total antioxidant capacity (millimolar of uric acid equivalents), ROS/RNS production (micromolar of DCF/L), and AOB index (TAC/ROS-RNS), and the cytokine profile evaluated by IL-6 (ng/mL), IL-1β (ng/mL), and IL-10 (ng/mL) expressed as the average of the fixed effects (feeding strategy and time of sampling).

	Fixed effects								
	Feeding strategy				Time of sampling				
	CON	HS	SEM	Value of *p*	7 days	35 days	56 days	SEM	Value of *p*
Oxidative and antioxidant status^1^									
DPPH, %	30.50^b^	37.01^a^	1.25	0.0005	29.20^b^	37.86^a^	34.20^ab^	1.54	0.0008
TAC, mM of uric acid eq.	0.13	0.13	0.001	0.195	0.12^b^	0.13^a^	0.13^a^	0.002	0.016
ROS/RNS, μM DFC/L	1230.26^b^	1498.97^a^	41.89	<0.001	1369.33	1328.65	1395.87	51.31	0.648
AOB	1.142	1.064	0.06	0.394	1.000	1.177	1.132	0.08	0.259
Cytokine profile									
IL-6, ng/mL	4.80	4.21	0.26	0.123	4.86^a^	4.92^a^	3.73^b^	0.33	0.0154
IL-1β, ng/mL	15.43^a^	12.33^b^	0.88	0.015	18.22^a^	12.35^b^	11.07^b^	1.08	<0.001
IL-10, ng/mL	3.81^b^	4.50^a^	0.15	0.002	3.50^b^	4.68^a^	4.28^a^	0.18	<0.001

^a,b^In the same row with various superscripts are significantly different (*p* < 0.05); CON = control maize–barley-based concentrated diet; HS = 15% of hazelnut skin (HS) as maize replacer of the concentrate diet at 15, 35, and 56 days; DPPH = 1,1-diphenyl-2-picrylhydrazyl; TAC = total antioxidant capacity; ROS/RNS = reactive oxygen species/reactive nitrogen species; DCF = 2′,7′-dichlorodihydrofluorescein; IL = interleukin.

The level of plasma TAC was affected by the time of sampling (*p* = 0.016), showing, on average, a higher TAC level at both 35 and 56 days than at 7 days (Table 1). The plasma ROS/RNS level showed a higher value in the HS group on average as an outcome of the feeding strategy (*p* < 0.001, Table 1). In particular, the tendency of the interaction effect between time of sampling and feeding strategy (*p* < 0.10) showed lower levels of this parameter in CON at 35 days than in the HS group at 7, 35, and 56 days of the experimental diet (Figure 1B). When TAC and ROS/RNS levels were included in the AOB index, no significant differences emerged (*p* = 0.394 for feeding strategy, and *p* = 0.259 for the time of sampling; respectively, Table 1).

### 3.2 Cytokine secretion

Plasma IL-6 secretion was significantly affected by time of sampling (*p* = 0.015), showing a significant decrease from 7 and 35 days to 56 days (Table 1).

**Figure 2:**
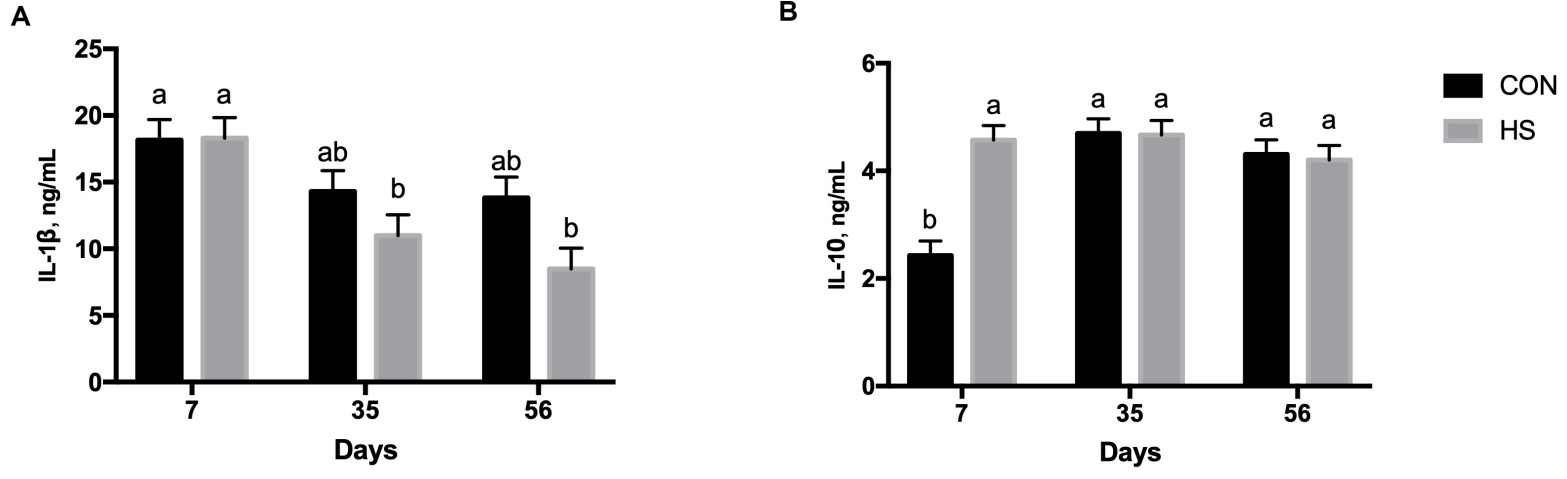
Plasma IL-1β **(A)** and IL-10 **(B)** secretion in lambs fed with a control maize–barley-based concentrated diet (CON) and with 15% of hazelnut skin (HS) as a maize substitute for the concentrate diet at 15, 35, and 56 days. a,b indicate significant differences among feeding treatments and time of sampling (*p* < 0.10 IL-1β, and for *p* < 0.05 for IL-10, respectively).

The secretion of IL-1β was significantly influenced by time of sampling (*p* < 0.001), exhibiting a lower level at 35 days and 56 days than that at 7 days, respectively (Table 1). Moreover, feeding strategy significantly influenced the IL-1β level (*p* = 0.015) with a higher value registered in the CON group than the HS group (Table 1). A tendency of the interaction effect between time of sampling and feeding strategy was registered (*p* < 0.10), showing a higher value of IL-1β in both experimental groups and at 7 days of the experiment than in the HS group as the lambs aged (Figure 2A).

Plasma IL-10 secretion was significantly affected by time of sampling (*p* < 0.001), feeding strategy (*p* = 0.002), and the interaction between time of sampling and feeding strategy (*p* < 0.001). The level of IL-10 was lower at 7 days of the experiment than at both 35 and 56 days (Table 1). Lambs from the HS group had a higher IL-10 plasma level than the CON group, on average (Table 1). The CON group had a lower level of IL-10 at 7 days than the HS group and than the level found in both experimental groups at 35 and 56 days (Figure 2B).

## 4 Discussion

To the best of our knowledge, no previous studies have investigated the role of hazelnut skin supplementation on the plasma antioxidant status and cytokine profiles of growing lambs.

In a recent comprehensive review by Zhao et al. (4), the authors underlined the multiple biological functions of hazelnut and its by-products that are linked to many health benefits due to the nutrients and phytochemicals. The HS by-product is rich in vitamin E and tannins, mainly condensed, with strong antioxidant properties that can be measured as scavenging free radicals using different methods, including DPPH (4). In the present study, an increase in plasma free radical scavenging activity, evaluated by DPPH assay, is registered in the HS group. This result is consistent with that of a previous study (12), in which the lamb’s plasma proteome profile is characterized by proteins involved in the antioxidant pathway. In line with this finding, it was found that grape pomace by-product supplementation, which is rich in polyphenols, causes a boosting effect on antioxidant mechanisms, especially glutathione, with overall protection against lipid peroxidation and protein oxidation, suggesting the enhancement of welfare in growing lambs (24). Moreover, in lambs infected with *Haemonchus contortus*, selenium supplementation, which is a component of the glutathione peroxidase (GSH-Px) enzyme (25), contributes to the antioxidant protection against oxidative stress, causing lower lipid peroxidation and higher blood GSH-Px activity (26). Therefore, it could be hypothesized that the higher radical scavenging activity of HS in plasma may be linked to the endogenous antioxidant system (27) mediated by the GSH-Px activity. When the antioxidant status of plasma was measured with the TAC assay, no significant differences were detected between the experimental groups. This result could be explained by the different structure–activity relationships for each antioxidant assay due to the dominant reaction, which depends on the pH and solvent used (28). Specifically, the DPPH radical reacts via the sequential proton loss electron transfer-SPLET mechanism in solvents such as ethanol and methanol (29, 30), while the TAC assay quantifies the capability of an antioxidant to transfer one electron to reduce any compound via the single electron transfer (SET) mechanism.

At an early age, lambs are exposed to several stressful conditions, such as weaning and adapting to a new feeding pattern, which occur in a gradual metabolic status stabilization and cause an immune and antioxidant system decline, thereby exposing animals to oxidative stress (31). In the present study, the increased level of ROS found in the HS group after 35 days of the experiment could be justified by the gradual adaptation to the tannin supplementation; however, the same group registers a high level of DPPH after 7 days of the experiment, supporting a gradual oxidative stabilization throughout the experiment. This statement is corroborated by the AOB (TAC/ROS-RNS) value as a measure of redox homeostasis, which does not result in any differences between the CON and HS groups, thus demonstrating the importance of monitoring both the antioxidant and oxidant status to evaluate oxidative stress exposure in growing lambs. The AOB index has been proposed as a reliable index of redox homeostasis in the plasma of heat-stressed sheep (18) and in human studies to evaluate the effects of diet on the serum antioxidant status (32–34), which underlines the need for a gold standard measure of the antioxidant status of sheep plasma (18).

Dietary antioxidants can influence inflammatory responses indirectly by controlling the antioxidant defense or interfering with oxidative stress signaling or directly by exerting a suppressive action on pro-inflammatory signaling transduction (35). The phenolic compounds that possess antioxidant activity are able to modulate the inflammatory response with suppressive action on pro-inflammatory signaling transduction (35) and on the Th1-type immune response (36). In accordance with this finding, the HS supplementation decreased the level of IL-1β, confirming the suppressive action of phenolic compounds on the pro-inflammatory status of plasma. Murine macrophage cell lines treated with resveratrol, a polyphenolic compound naturally produced by several plants, inhibit NLRP3 (NACHT, LRR, and PYD domains containing protein 3) inflammasome-derived IL-1β secretion, reducing mitochondrial damage and increasing autophagy (37). Sheep peripheral blood mononuclear cells treated with wine lees from white grape vinification reduce the secretion of IL-1β without affecting the level of IL-6 (38). Accordingly, the HS supplementation does not modify the secretion of IL-6; however, its level decreased along with the experimental trial. Interleukin-6 is defined as a cytokine with different biological functions that depend on the function and structure of its receptor (39). IL-6 production is triggered in response to bacterial and viral infections and other cytokines such as IL-1β, TNF, and IFN-γ (40). Therefore, the high level of IL-1 β and the absence of inflammatory challenge can help justify the absence of differences in IL-6 secretion among experimental groups. In addition, the modulatory role of HS could be due to its composition in terms of fatty acids, as demonstrated by the increased stearic (C18:0), oleic (C18:1 c9), palmitic (C16:0), and linoleic acid (C18:2 c9, c12) fatty acids ingested by HS lambs (9). Therefore, the presence of these fatty acids could modulate the inflammatory responses of growing lambs, as previously demonstrated in growing calves (41).

Interleukin-10 is defined as a regulatory cytokine (42), which can act in innate as well as adaptive immunity, both in terms of immunosuppressive and immunostimulatory effects in many cell types (43), especially during the resolution phase of inflammation (44). In this context, IL-10 is able to inhibit the production of several inflammatory cytokines, such as TNF-α, IL-1β, and IL-6, and it is described as the key antagonist of the Th1 response (45–47). In the present study, lambs supplemented with HS show a regulatory profile on cytokine secretion, which results in an increase of IL-10 and a decrease of IL-1β, indicating an anti-inflammatory effect of hazelnut skin’s phenolic compounds. In line with our results, the tannic acid is able to modulate the immune responses of whole blood cells in sheep stimulated by *Haemonchus contortus* crude larval antigen by shifting the Th1 cytokines versus Th2 cytokine expression levels in cells (48). Consistently, in grazing dairy cows receiving supplementation with tannins (49), a suppressive action of tannins on IL-1β Th1 cytokines coupled with the increased level of IL-10 is found. Taking into account data on the cytokines’ profile of growing lambs, a role for HS in modulating cytokine secretion in favor of the regulatory/anti-inflammatory IL-10 could be assumed. Present evidence supports the use of HS as a feed supplement in lambs’ diets and underlines the need to further investigate the role of by-products in immune responses as a part of the more complex system connected to animal welfare in sustainable livestock production. Moreover, a possible role of condensed tannins in the improvement of the endogenous antioxidant system could be hypothesized with the implication of GSH-Px activity, which needs to be further elucidated in growing lambs.

## 5 Conclusion

The present experiment demonstrated the antioxidant and anti-inflammatory role of HS as a 15% maize substitute in the lambs’ concentrate diet. In particular, the lambs’ plasma free radical scavenging activity resulted in an increase in the HS group, with a concomitant increase in ROS production in comparison with the CON group. The redox homeostasis measured with the AOB index confirmed the balance between the total antioxidant capacity of plasma and ROS/RNS production in both experimental groups. The cytokines measured in plasma exhibited an anti-inflammatory/regulatory profile with high levels of IL-10 and low levels of IL-1β in lambs supplemented with HS, which can be useful to counteract an inflammatory condition linked to the lambs’ growing phase.

## Data availability statement

The raw data supporting the conclusions of this article will be made available by the authors, without undue reservation.

## Ethics statement

The animal study was approved by the Ethical and Animal Wellbeing Commission of the University of Catania (protocol code 15295). The study was conducted in accordance with the local legislation and institutional requirements.

## Author contributions

MGC: Data curation, Methodology, Writing – original draft. AnS: Conceptualization, Data curation, Investigation, Supervision, Validation, Writing – original draft. MC: Data curation, Validation, Writing – review & editing. AdM: Methodology, Writing – review & editing. AN: Methodology, Writing – review & editing. AB: Methodology, Writing – review & editing. MA: Conceptualization, Data curation, Supervision, Validation, Writing – original draft. AgS: Conceptualization, Investigation, Project administration, Supervision, Validation, Visualization, Writing – review & editing.
